# Enzymatic Formulation Capable of Degrading Scrapie Prion under Mild Digestion Conditions

**DOI:** 10.1371/journal.pone.0068099

**Published:** 2013-07-16

**Authors:** Emeka A. Okoroma, Diane Purchase, Hemda Garelick, Roger Morris, Michael H. Neale, Otto Windl, Oduola O. Abiola

**Affiliations:** 1 Department of Natural Sciences, School of Science and Technology, Middlesex University, London, United Kingdom; 2 School of Biomedical Sciences, King’s College London, London, United Kingdom; 3 Animal Health and Veterinary Laboratories Agency (AHVLA), Surrey, United Kingdom; 4 PAP Rashidah Sa’adatul Bolkiah Institute of Health Sciences, Universiti Brunei Darussalam, Gadong, Brunei Darussalam; Deutsches Zentrum für Neurodegenerative Erkrankungen e.V., Germany

## Abstract

The prion agent is notoriously resistant to common proteases and conventional sterilisation procedures. The current methods known to destroy prion infectivity such as incineration, alkaline and thermal hydrolysis are harsh, destructive, environmentally polluting and potentially hazardous, thus limit their applications for decontamination of delicate medical and laboratory devices, remediation of prion contaminated environment and for processing animal by-products including specified risk materials and carcases. Therefore, an environmentally friendly, non-destructive enzymatic degradation approach is highly desirable. A feather-degrading *Bacillus licheniformis* N22 keratinase has been isolated which degraded scrapie prion to undetectable level of PrP^Sc^ signals as determined by Western Blot analysis. Prion infectivity was verified by *ex vivo* cell-based assay. An enzymatic formulation combining N22 keratinase and biosurfactant derived from *Pseudomonas aeruginosa* degraded PrP^Sc^ at 65°C in 10 min to undetectable level -. A time-course degradation analysis carried out at 50°C over 2 h revealed the progressive attenuation of PrP^Sc^ intensity. Test of residual infectivity by standard cell culture assay confirmed that the enzymatic formulation reduced PrP^Sc^ infectivity to undetectable levels as compared to cells challenged with untreated standard scrapie sheep prion (SSBP/1) (*p*-value = 0.008 at 95% confidence interval). This novel enzymatic formulation has significant potential application for prion decontamination in various environmentally friendly systems under mild treatment conditions.

## Introduction

Transmissible Spongiform Encephalopathies (TSEs) or prion diseases such as Creutzfeldt-Jakob disease (CJD) in humans, Bovine Spongiform Encephalopathy (BSE) in cattle, Chronic Wasting Disease (CWD) in mule deer and elk and scrapie in sheep and goat are a group of closely related, progressive, incurable and invariably fatal neurodegenerative disorders that affect the central nervous system (CNS) of mammals [1], [2].

The TSE agent is resistant to common proteases and withstands conventional physical and chemical sterilisation, inactivation and decontamination procedures [3]-[6].

Common prion decontamination methods includes porous load autoclaving at 134°C for 18 min and immersing contaminated material in 1 M NaOH and/or 20,000 ppm NaOCl for 1 h at 20°C [1], [7]. However, these methods result in irreversible damage to medical devices [8], and may be ineffective in destroying prion infectivity [5], [6], [9].

Incineration [10], [11], thermal hydrolysis [12] and alkaline hydrolysis [13]-[15] destroy prions effectively. While incineration is suitable for destruction of prion in non-recoverable materials, it is impractical for the decontamination of recoverable and reusable materials (e.g. animal by-products), delicate medical and laboratory instruments, and contaminated environment [16]. Residual infectivity has also been reported after incineration of scrapie contaminated tissue [17]-[19]. In addition, environmental impact concerns, regulatory requirements and cost of such facility are limiting factors. Alkaline hydrolysis is unsuitable for application in the rendering of recoverable Specific Risk Materials (SRM) and carcases of prion infected tissue as it could result in end products that are extremely degraded and contain high concentration of salt residue, therefore diminishing its commercial value [20].

Enzymatic (microbial) degradation of prions has been explored for the obvious advantages that it is potentially able to destroy prion infectivity without destroying the decontaminated material. It is also the most practicable approach for remediation of prion contaminated environment without adversely affecting the environmental biota and/or ecology [21]–[25].

Since the earliest report of enzymatic degradation of scrapie prion [26], there have been several studies investigating prion decontamination [22], [27]–[33]. In most cases, the prion substrate was rendered proteolyticly susceptible by either pre-heating at high temperature, digesting in the presence of chemical surfactants/detergents, denaturants and oxidising agents, incubating under high alkaline conditions and digesting for an extended period of time or a combination of these approaches. The importance of detergents in the enzymatic degradation mechanism, such as in solubilising the prion substrate, has been particularly highlighted [22], [29], [30].

In general, the established methods of enzymatic degradation are harsh, environmentally and economically unsustainable, and may require a sequential multi-step approach that is complex and impractical for commercial application. Therefore, a simple, effective and efficient enzymatic degradation method that combines moderate pH and temperature conditions, relatively short digestion time and biological surfactant (biosurfactant) is highly desirable.

Biosurfactants are extracellular or membrane-associated amphiphilic surface-active biomolecules derived from biological sources [34], [35]. *Pseudomonas aeruginosa* is one of the prolific producers of biosurfactants [36], [37]. Biosurfactants are composed of a hydrophilic (head) and hydrophobic (tail) moieties [38], [39]. The hydrophilic moiety is usually a carbohydrate, amino acid, phosphate, cyclic peptide, carboxylic acid, or alcohol and the hydrophobic moiety is mostly a long-chain fatty acid, or fatty acid derivatives such as hydroxyl fatty acid or α-alkyl-β-hydroxy fatty acid.

Biosurfactants have various applications in the petroleum industry [38], [40], environmental bioremediation [41]-[43], agricultural biocontrol [44]-[46] and in cosmetic, pharmaceutical and therapeutic products industry [35], [39], [47].

This paper reports the degradation of scrapie prion under mild digestion conditions by a novel enzymatic formulation which comprises keratinase and biosurfactant isolated from bacterial sources.

## Materials and Methods

### Materials

#### Brain homogenate

Harvested whole brain was homogenized in PBS into 10% (w/v) brain homogenate and stored at −80°C. Subsequently, 1% (w/v) brain homogenate was prepared in sterile phosphate-buffered saline (PBS) to be used for digestion experiments. ME7 brain homogenate (kindly provided by Drs Stephen Whatley and Oduola Abiola of Neuroscience Department, KCL Institute of Psychiatry, London), SSBP/1 sheep scrapie homogenate (kindly provided by the biological archive Animal Health and Veterinary Laboratories Agency, UK) were used as scrapie brain homogenate materials, and normal brain homogenate was derived from C57BL/6 mice.

ME7 brain homogenate was produced under the UK Home Office Project Licence: ‘Host genetics of prion disease transmission’ and approved by King’s College London Ethical Review Process Committee (Denmark Hill Campus). Brain homogenate from C57BL/6 mice was covered by the licence PPL70/6760: ‘How cells and tissues produce and respond to neurodegenerative amyloid’ and approved by King’s College London Ethical Review Process Committee (Guy’s Campus). The SSBP/1 material was produced under the Home Office Project Licence no. 70/5780 and was approved by the AHVLA Ethical Review Committee. All work fully met the requirements of the UK Animals (Scientific Procedures) Act 1986. All infectious scrapie material was handled according to the World Health Organisations infectious control guideline for transmissible spongiform encephalopathies [1].

#### Cell line and culture medium

SSBP/1-susceptible RK13^VRQ.G9^ cells were engineered to constitutively express ovine VRQ PrP allele; RK13 was transfected with a vector containing the ovine VRQ PrP gene under the control of the CMV promoter. The vector was described in a paper by Piening et al. [48]. RK13^VRQ.G9^ cells have similar level of sensitivity as Rov9 cells [49] to SSBP/1 infection (Neale, unpublished data) and were used in the standard scrapie cell assay (SSCA). The cells were maintained in Eagle’s minimal essential medium (EMEM, Gibco) supplemented with 10% fetal calf serum and 2% HEPES. RK13^VRQ.G9^ infection was cultured in OptiMEM medium (Invitrogen) supplemented with 10% foetal calf serum (Gibco) and antibiotics/antimyotic (Penicillin, 100 units; Streptomycin, 100 µg and Amphotericin B, 0.25 µg, Invitrogen).

#### Proteases and biosurfactant

Bacillus licheniformis N22 keratinase (EF) and Pseudomonas aeruginosa NCIMB 8626 biosurfactant (BS) were isolated as previously described [50] and [51] respectively. An enzymatic formulation (EF+BS) composed of keratinase and biosurfactant was prepared in the laboratory at Middlesex University. Proteinase K (PK) was bought from Fisher scientific, UK.

#### Assay antibodies

SAF83 (SPI-Bio, France) and Sha31 (Bertin Pharma) were used as primary antibodies to detect PrP signal in the Western Blot analysis. The secondary antibodies used were the Amersham ECL sheep anti-mouse IgG Horseradish peroxidase linked whole antibody (GE Healthcare, UK) and goat anti-mouse alkaline phosphatase conjugate (Sigma, UK).

### Methods

#### Digestion of brain homogenate substrates

10 µl of 1% scrapie-infected brain homogenates (IBH) was digested with PK (1 µl), EF (1 µl), BS (1 µl) or EF+BS at pH7, digestion times (ranging from 10 min to 2 h) at 50°C or 65°C as specified for each experiment. Time-course digestion with EF+BS was carried out for 30, 45, 60, 90 and 120 min at 50°C, and for 10, 30 and 45 min at 65°C. EF (0.05, 0.1 and 0.4 µg/ml), BS (75 µg/ml) and PK (10, 50 and 100 µg/ml) final concentrations were investigated to determine their optimum concentrations. PK-digested and undigested IBH were used as the positive and the negative controls respectively. Digestion reaction was stopped with 1 µl of 50 mM Phenyl methyl sulfonyl fluoride (PMSF). All digestions were carried out in triplicates.

#### Western Blot Analysis

Digested samples were mixed with 2× sample buffer (11 µl) and heated for 10 min at 100°C on a dry block (Techne, UK). Samples (10 µl) were electrophoresed on 12% gel and the separated proteins were transferred onto polyvinylidene fluoride (PVDF) membrane by Trans-Blot SD semi-dry transfer cell (Biorad). The membrane was blocked with 5% skimmed milk for 1 h, and then probed with SAF 83 monoclonal antibody (1∶5000) for 1 h while shaking at 30 rpm. The membrane was washed twice with 0.1% Tween in 1× PBS (PBST) and a further six washes at 5 min intervals. It was then probed with sheep anti-mouse IgG Horseradish peroxidase linked whole antibody (secondary antibody) at 1∶5000 dilutions and incubated at room temperature for 50 min, shaking at 30 rpm and washed as described above. PrP signal was detected with Amersham™ ECL™ Plus Western Blot Detection System (GE Healthcare, UK) as described by the manufacturer. The image was photographed on Kodak film, and developed with Mediphot 937 X-ray film processor (Colenta Labortechnik, Austria). Each replicated digested sample was loaded and run on gels for three separate times.

#### Standard scrapie cell culture assay (SSCA)

The SSCA was performed at Animal Health and Veterinary Laboratories Agency, New Haw, Weybridge, UK as previously described [52], with the only difference being that RK13^VRQ.G9^ cells was used as opposed to Rov9 and MovS6 cells, and Sha31 was used instead of 6H4 as the primary antibody. Five different treatments groups were investigated: IBH (cells inoculated with neat SSBP/1), Incubated IBH (cells inoculated with SSBP/1 that was incubated at the digestion temperature), EF (Cells inoculated with N22 keratinase-digested SSBP/1), BS (cells inoculated with biosurfactant-digested SSBP/1) and EF+BS (cells inoculated enzymatic formulation-digested SSBP/1). All sample digestions were carried out at 65°C for 1 h. The number of positive cells (spots) were counted using Zeiss KS-ELISPOT imaging system running Wellscan software. All experiments were repeated in quadruplet.

#### Statistical analysis

Test of difference in the number of infected cells in the treatment groups were compared using Two-Sample T-Test.

## Results

### In vitro Degradation of ME7 Scrapie

**Figure 1:**
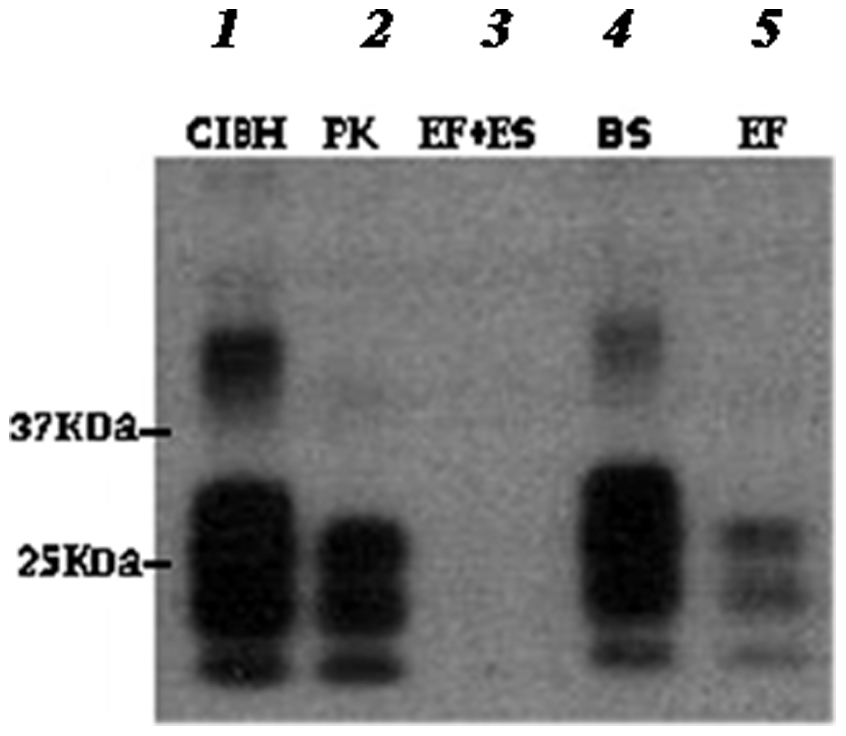
Western blot profile of ME7 brain homogenate digested with EF with and without BS. Lanes 2, 3, 4, and 5 are ME7 scrapie brain homogenate digested at 65°C for 1 h with PK (100 µg/ml), EF+BS, BS and EF respectively. Total removal of PrP^Sc^ signal was achieved with EF+BS only. PrP^Sc^ was probed with SAF83 mAb.

ME7 scrapie brain homogenate (10 µl) digested with the enzymatic formulation (EF+BS) at 65°C for 1 h resulted in undetectable level of PrP^Sc^ as determined by Western Blot analysis (Fig. 1; Lane 3). Under these conditions, keratinase (EF) alone was unable to completely degrade ME7 scrapie, resulting in the typical PrP^Sc^ glycosylation bands (Fig. 1; Lane 5). Biosurfactant (BS) alone showed no discernible activity towards PrP^Sc^ degradation (Fig. 1; Lane 4). Digestion with PK resulted in the typical PrP^res^ bands (Fig. 1; Lane 2).

**Figure 2:**
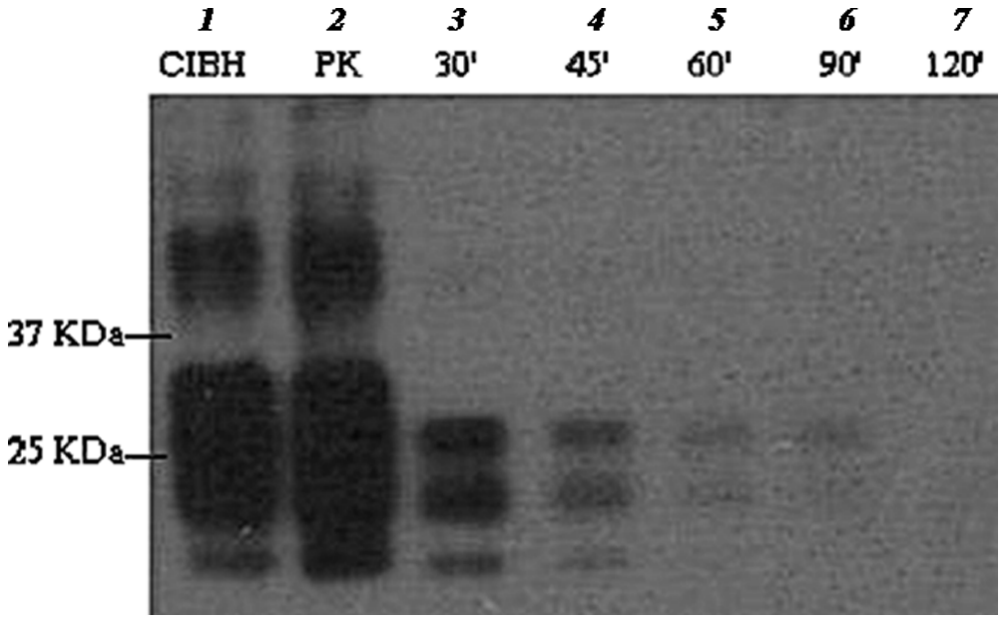
Time-course profile of ME7 brain homogenate digested with EF+BS at 50°C. Lane 1 is neat ME7 brain homogenate (positive control) and lane 2 is proteinase K treated sample (77 µg/ml final PK conc.). Lanes 3–7 are digested with EF+BS at 30–120 min respectively. Samples were digested at 50°C and PrP^Sc^ was probed with SAF83 mAb.

**Figure 3:**
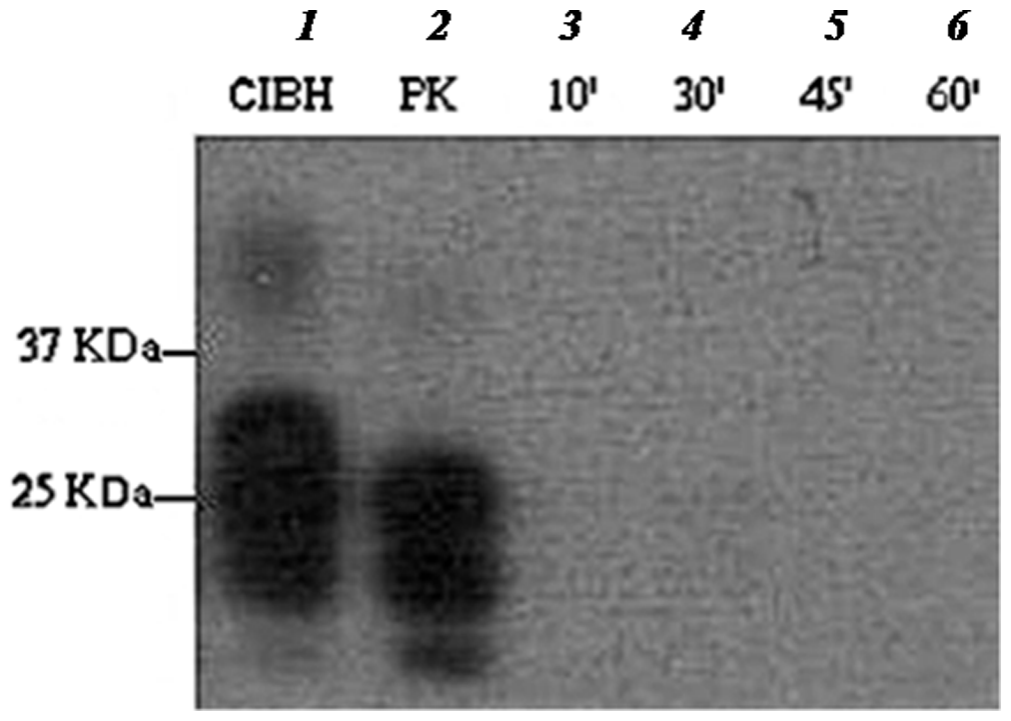
Time-course profile of ME7 brain homogenate digested with EF+BS at 65°C. Lane 1 is neat ME7 brain homogenate (positive control) and lane 2 is proteinase K digested sample (77 µg/ml final PK conc.). Lanes 3–6 are digested with EF+BS at 10, 30, 45 and 60 min. Samples were digested at 65°C and PrP^Sc^ was probed with SAF83 mAb.

Time-course degradation carried out at 50°C for 30, 45, 60, 90 and 120 min demonstrated the progressive loss of detectable PrP^Sc^ signal over time (Fig. 2). Further optimisation showed complete degradation of PrP^Sc^ down to undetectable levels in 10 min at 65°C (Fig. 3).

**Figure 4:**
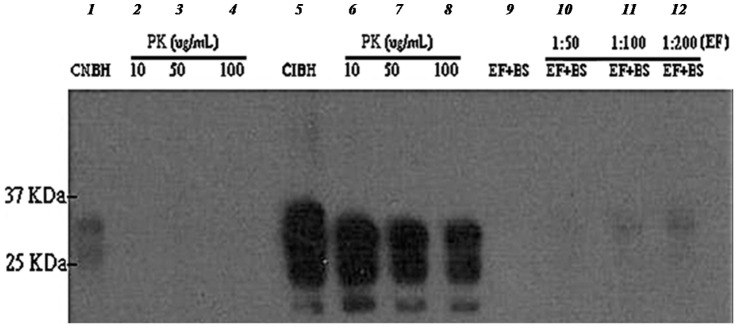
Digestion of normal and ME7 brain homogenates with different concentrations of PK and EF. Normal (NBH) and infected (IBH) brain homogenates digested at 65°C for 1 h with 10, 50 and 100 µg/ml of proteinase K (lanes 2, 3, 4 and lanes 6, 7, 8 respectively) and EF+BS formulation [1∶50, 1∶100 and 1∶200 dilutions or 0.4, 0.1 and 0.05 µg/ml of EF and 75 µg/ml rhamnose equivalent (BS)] (lanes 9, 10, 11, 12) probed with SAF83 monoclonal antibody. Lanes 1 and 5 are undigested NBH and IBH controls, respectively.

PK at final concentrations of 10, 50 and 100 µg/ml completely digested normal brain homogenate (NBH) to undetectable levels of PrP (Fig. 4; lanes: 2–4) but did not digest ME7 infectious brain homogenate (IBH) beyond the expected reduction in size due to removal of the N-terminal domain (Fig. 4; lanes: 6–8). Digestion with the enzymatic formulation (EF+BS) at final concentrations of EF (0.05, 0.1 and 0.4 µg/ml) and BS (75 µg/ml rhamnose equivalent) resulted in complete or nearly complete loss of PrP^Sc^ signal in order of increasing dilution (Fig. 4).

### Scrapie Cell Assay

To confirm the efficacy of the enzymatic formulation (EF+BS) to completely eliminate prion infectivity, SSBP/1 susceptible RK13^VRQ.G9^ cells were challenged with enzyme-digested SSBP/1 scrapie and analysed for residual infectivity.

**Figure 5:**
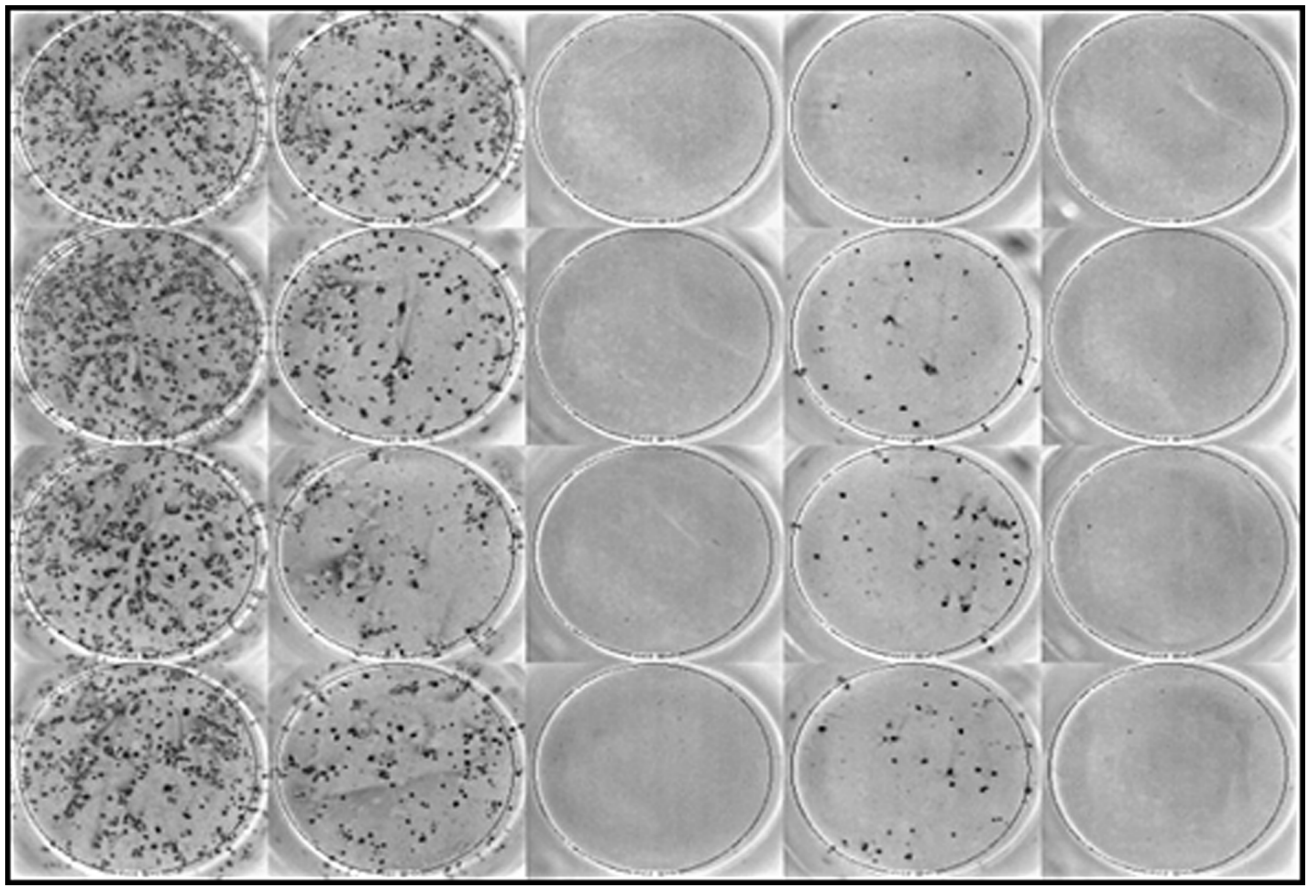
Representative photograph of ELISPOT plate. The wells contained infected RK13^VRQ.G9^ cells inoculated with IBH (SSBP/1), incubated IBH (heat treated SSBP/1) and BS (Biosurfactant digested SSBP/1). Wells inoculated with SSBP/1 digested with EF and EF+BS were completely devoid of infected cells. PrP^Sc^ was probed with sha31 mAb.

**Figure 6:**
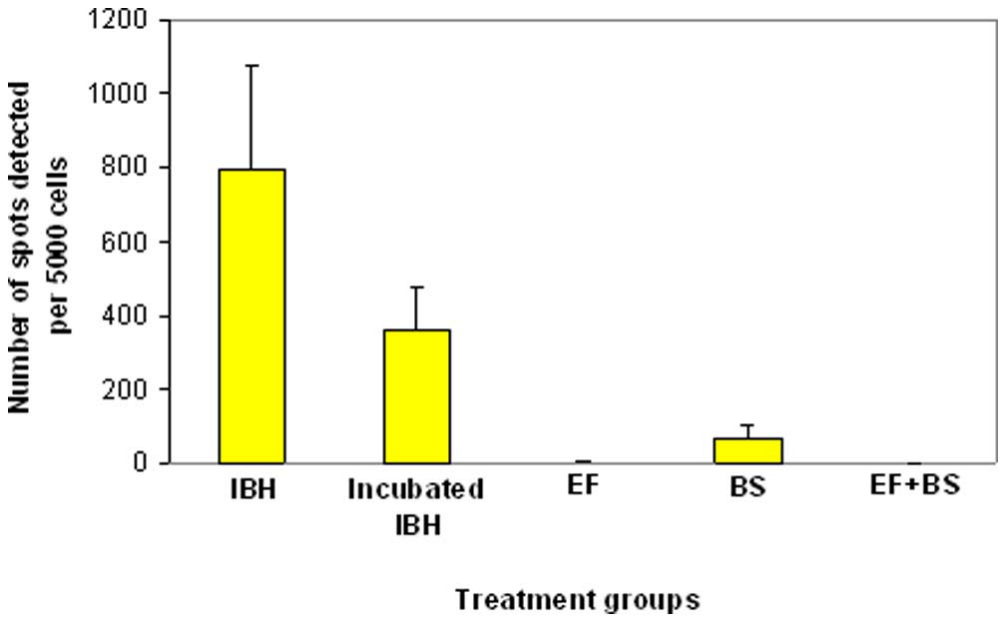
Number of infected cells (spots) for the different treatment groups. Treatment groups: IBH (inoculated with neat SSBP/1), Incubated IBH (inoculated with SSBP/1 that was incubated at the digestion temperature), EF (inoculated with keratinase-digested SSBP/1), BS (inoculated with biosurfactant-digested SSBP/1) and EF+BS (inoculated with enzymatic formulation-digested SSBP/1). The number of infected cells were significantly reduced for EF (*p* = 0.009), BS (*p* = 0.033) and EF+BS (*p* = 0.009) treatment groups compared to Incubated IBH. Test of significance was calculated using Two-Sample T-Test. Each bar represents mean ± SD (n = 4, each data point).

Prion infectivity study confirmed that RK13^VRQ.G9^ cells propagated SSBP/1 scrapie prion but RK13^VRQ.G9^ cells inoculated with EF and EF+BS digested SSBP/1 scrapie did not propagate infectivity as shown by the absence of infected cells (detectable spots in the ELISPOT assay: Fig. 5). The number of infected cells per 5000 cells (cell number confirmed by trypan blue assay) were significantly lower for both the EF and EF+BS treatment groups compared to the incubated IBH group (both have *p* = 0.009, Two-Sample T-Test; Fig. 6). Although, a significantly lower number of cells were infected in the BS treatment group compared to the incubated IBH group (*p* = 0.033, Two-Sample T-Test; Fig. 6), the results show that cells inoculated with BS-treated brain homogenate sample remained infectious and were able to propagate infection in RK13^VRQ.G9^ cells. It was also noted that SSBP/1 infectivity was significantly reduced by incubation at 65°C compared to IBH that was not exposed to heating (*p* = 0.044, Two-Sample T-Test; Fig. 6).

## Discussion

### Degradation of ME7 Scrapie Prion

The enzymatic formulation (EF+BS) partially degraded ME7 scrapie prion at 50°C in 1 h but when the temperature was raised to 65°C, it was efficiently degraded to levels undetectable by Western blot analysis in a short digestion time of 10 min (Fig. 3). This clearly indicates that there was a significant thermal effect on ME7 prion degradation. Keratinase (EF) alone could not fully digest ME7 prion at 65°C in 1 h compared to complete digestion with EF+BS. This suggests a significant role of biosurfactant (BS) in the degradation mechanism. Therefore, the interplay of digestion time, temperature and biosurfactant is crucial in enzymatic prion degradation. In addition, significant level of PrP^Sc^ degradation at low concentration of N22 keratinase (EF) (0.05 µg/ml) demonstrates the efficacy of this enzyme (Fig. 4).

ME7 scrapie has been reported to be thermally stable up to 84°C [53]. In this study, digestion at 65°C may not have independently destabilised ME7 scrapie molecular structure but appears to be a critical factor in the ME7-EF-BS reaction complex. The catalytic activity of the keratinase and the solubilising property of biosurfactant were central in the degradation mechanism, which may also involve PrP^Sc^ structure unfolding, weakening of disulphide bonds, proteolytic access and cleavage of structural bonds as well as substrate solubilisation as have been previously suggested [22], [54], [55].

BS on its own could not degrade ME7 scrapie suggesting that it lacked proteolytic activity and prion degrading potential. Some *Pseudomonas* strains have been found to produce keratinases [56], [57] and a *Bacillus licheniformis* strain have been reported to concomitantly produce alkaline protease and biosurfactant [58], but the biosurfactant of *Pseudomonas aeruginosa* NCIMB 8626 investigated in this study lacked keratinase activity and *Bacillus licheniformis* N22 keratinase lacked biosurfactant properties. Therefore, in this study EF and BS appear to play different but specific roles, forming a remarkable synergistic effect in the enzymatic degradation process.

In the time-course experiment, the loss of the PrP band due to removal of the N-terminal domain suggests the cleavage of PrP^Sc^ by the enzymatic treatment, and further illustrates the interplay of incubation time and temperature in the prion degradation process. Therefore, the decision to choose a shorter incubation time over higher temperature or vice versa will depend on the specific application need (e.g. decontamination of sensitive surgical device and remediation of prion contaminated soil) and economic consideration.

### Standard Scrapie Cell Assay (SSCA)

Although Western blot analysis is a commonly used biochemical method for prion immunodetection, the presence of prion infectivity in samples with the apparent lack of detectable levels of PrP^Sc^ [59]-[61] limits its use for evaluating residual prion infectivity. A more consistent, reliable and sensitive method such as the SSCA was required to confirm results from Western blot analysis and to validate the efficacy of the enzymatic formulation for prion degradation.

SSCA was carried out to establish if the absence of PrP^Sc^ detection in the Western blot profile of the enzyme-digested SSBP/1 was accompanied by corresponding loss in prion infectivity. The result of the SSCA confirmed that the enzymatic formulation efficiently destroyed prion infectivity as shown by the inability of the enzyme-digested SSBP/1 brain homogenate to infect susceptible RK13^VRQ.G9^ cells (Figs 5 and 6). While EF also destroyed SSBP/1 infectivity, BS resulted in a significant reduction in the number of infected cells which suggests that SSBP/1 infectivity was significantly attenuated at the digestion temperature (65°C) but not enough to prevent infection of cells. The number of spot counts detected in the EF and enzymatic formulation treatment groups were probably background noise as confirmed by absence of visually detectable infected cells on the photographic image of the ELISPOT plate (Fig. 5).

The inability of the enzyme-digested scrapie substrate to propagate infectivity in the susceptible RK13^VRQ.G9^ cells suggests the complete loss of SSBP/1 infectivity. Therefore, the efficacy of this enzymatic formulation for complete destruction of prion infectivity has been validated by the SSCA. This result suggests that the loss of detectable levels of PrP^Sc^ signal in the enzyme-digested scrapie prion as determined by Western blot correlated with loss of prion infectivity as determined by the SSCA. The complete destruction of the infectivity of field isolate of sheep scrapie (SSPB/1) is particularly very important in terms of disposal of sheep scrapie.

### Potential Applications of this Enzymatic Treatment Method

Hitherto, the inability to achieve efficient prion degradation at mild digestion conditions (neutral pH, moderate temperature, and low enzyme concentration) limits the use of enzymatic decontamination in economic and operational terms. Therefore, enzymatic degradation at mild digestion conditions is of general interest in the decontamination of sensitive medical devices, animal products [Meat and Bone Meal (MBM) and SRM] and prion contaminated environment.

The enzymatic degradation method described in this study degraded scrapie prion under moderate physical condition (pH 7 and 65°C) and digestion time (10 min). In addition, it did not require a truncated multi-step approach (e.g. [22], [29], [45]) in which the infectious material is either pre-treated and/or digested with multiple enzymes or in the presence of chemical surfactants or alkali carrier such as NaOH. This enzymatic treatment method promises to be efficient and practical, and the constituent agents (N22 keratinase and biosurfactant) are purely biological agents of potentially low production cost. Thus, this method could provide a good, environmentally friendly, more economically viable and safe alternative to existing prion decontamination methods.

Further work will include the decontamination of steel and soil-bound prion with this enzymatic preparation.

### Use of Biosurfactant in Prion Degradation

Chemical surfactants/detergents enhance enzymatic prion degradation [22], [26], [29], [31], [62], [63]. However, biosurfactants are particularly useful and advantageous because of their environmentally friendly properties (e.g. low toxicity, high biodegradability) and their biochemical properties enabling lowering of surface tension, increasing substrate surface area, substrate solubilisation and protein unfolding [32], [64]. In addition, biosurfactants inhibit pathogen adhesion and formation of biofilms on steel surfaces [35], [46], presenting a two fold advantage for decontamination of steel surfaces such as medical devices. Using biosurfactant would also prevent undesirable chemical load in the enzymatic degradation processes, and the need for additional facilities for effluent treatment.

This is the first report on the use of biosurfactant in the degradation of prion. In this study, crude biosurfactant was used resulting in substantial saving in cost and labour associated with biosurfactant purification.

## Conclusion

A novel enzymatic formulation combining keratinase and biosurfactant in a remarkable synergy which efficiently degraded scrapie prion and destroyed its infectivity has been reported. This system has great potential for use in the environmentally friendly prion decontamination processes of surgical instruments and recoverable materials in general.

## References

[1] World Health Organisation (1999) WHO infection control guidelines for transmissible spongiform encephalopathies. Report of a WHO Consultation. Geneva, Switzerland: WHO. Available: http//www.who.int/csr/resources/publications/bse/en/whocdscsraph2003.pdf. Accessed March 2009.

[2] Collinge J (2001) Prion diseases of humans and animals: their causes and molecular Basis. Annu Rev Neurosci 24: 519–550. 11283320 10.1146/annurev.neuro.24.1.519

[3] Taylor DM, Fraser H, McConnell I, Brown DA, Brown DL, et al (1994) Decontamination studies with the agents of bovine spongiform encephalopathy and scrapie. Arch Virol 139: 313–326. 7832638 10.1007/BF01310794

[4] Brown P, Gibbs CJ Jr, Amyx HL, Kingsbury DT, Rohwer RG, et al (1982) Chemical disinfection of Creutzfeldt-Jakob disease virus. N Eng J Med 306: 1279–1282. 10.1056/NEJM198205273062107 7040968

[5] McDonnell G, Burke P (2003) The challenges of prion decontamination. Clin Infect Dis 36: 1152–1154. 12715310 10.1086/374668

[6] Edgeworth JA, Sicilia A, Linehan J, Brandner S, Jackson GS, et al (2011) A standardized comparison of commercially available prion decontamination reagents using the Standard Steel-Binding Assay. J Gen Virol 92: 718–726. 21084494 10.1099/vir.0.027201-0 PMC3081234

[7] Rutala WA, Weber DJ (2010) Guideline for disinfection and sterilization of prion contaminated medical instruments. Infect Control Hosp Epidemiol 31: 107–117. 20055640 10.1086/650197

[8] Brown SA, Merritt K, Woods TO, Busick DN (2005) Effects on instruments of the World Health Organisation recommended protocols for decontamination after possible exposure to transmissible spongiform encephalopathy contaminated tissue. J Biomed Mater Res Part B Appl Biomater 72B: 186–190. 10.1002/jbm.b.30125 15449256

[9] Taylor DM (1999) Inactivation of prions by physical and chemical means. J Hosp Infect 43: S69–S76. 10658760 10.1016/s0195-6701(99)90067-1

[10] Defra (2005) BSE: Public health issues - Notification, disposal & compensation. Available:http://www.defra.gov.uk/animalh/bse/publichealth/notification.html. Accessed 13 Feb. 2009.

[11] Jennette JP (2002) Medical Waste Management at Cornells College of Veterinary Medicine Available: http://www.cayugalake.org/newsletter/spring/2002/medicalwaste.html.Accessed Feb.2009.

[12] Somerville RA, Fernie K, Smith A, Andrews R, Schmidt E, et al (2009) Inactivation of TSE agent by a novel biorefinement system. Process Biochem. 44: 1060–1062.

[13] Thacker HL, Kastner J (2004) Alkaline hydrolysis. Carcass disposal: A comprehensive review. Available: http://fss.k-state.edu/FeaturedContent/CarcassDisposal/PDF%20Files/CH%206%20-%20Alkaline%20Hydrolysis.pdf. Accessed Jan. 2009.

[14] Kalambura S, Kricka T, Jukic Z, Voca N, Kalambura D (2005) Alkaline hydrolysis of slaughterhouse waste. Krmiva 47: 97–100.

[15] Yokoyama T, Shimada K, Tagawa Y, Ushiki YK, Iwamaru Y, et al (2006) Western blot assessment of prion inactivation by alkali treatment in the process of horticultural fertilizer production from meat meal. Soil Sci Plant Nutr 52: 71–76.

[16] Saunders SE, Burtett-Hunt SL, Bartz JC (2008) Prions in the environment. Ocurrence, fate and mitigation. Prion 2: 162–169. 19242120 10.4161/pri.2.4.7951 PMC2658766

[17] Brown P, Liberski PP, Wolff A, Gadjusek DC (1990) Resistance of scrapie infectivity to steam autoclaving after formaldehyde fixation and limited survival after ashing at 360°C: Practical and theoretical implications. J Infect Dis 161: 467–472. 2107265 10.1093/infdis/161.3.467

[18] Brown P, Rau EH, Lemieux P, Johnson BK, Bacote AE, et al (2004) Infectivity studies of both ash and air emissions from simulated incineration of scrapie-contaminated tissues. Environ Sci Technol 38: 6155–6160. 15575075 10.1021/es040301z

[19] Woodward JB (2004) Grand Junction TSE incinerator. Available: http://www.maddeer.org/saved/022404woodward.html. Accessed 20 January 2009.

[20] Coll BA, Garcia RA, Marmer WN (2007) Diffusion of protease into meat and bone meal for solubility improvement and potential inactivation of the BSE prion. PLoS ONE Available: http://www.plosone.org/article/info%3Adoi%2F10.1371%2Fjournal.pone.0000245. Accessed April 2011. 10.1371/journal.pone.0000245 PMC1796944 17327909

[21] Shih JCH (2002) Method and composition for sterilizing surgical instruments. US Patent Aplication. US 2002/0192731.

[22] Langeveld JPM, Wang JJ, Van De Wiel DFM, Shih GC, et al (2003) Enzymatic degradation of prion protein in brain stem from infected cattle and sheep. J Infect Dis 188: 1782–1789. 14639552 10.1086/379664

[23] Huang H, Spencer JL, Soutyrine A, Guan J, Rendulich J, et al (2007) Evidence for degradation of abnormal prion protein in tissues from sheep with scrapie during composting. Can J Vet Res 71: 34–40. 17193880 PMC1636000

[24] Rapp D, Potier P, Jocteur-Monrozier L, Richaume A (2006) Prion degradation in soil: possible role of microbial enzyme simulated by the decomposition of buried carcasses. Environ Sci Technol 40: 6324–6329. 17120560 10.1021/es060943h

[25] Saunders SE, Jason C, Bartz JC, Vercauteren CC, Bartelt-Hunt SL (2011) An enzymatic treatment of soil-bound prions effectively inhibits replication. Appl Environ Microbiol 77: 4313–4317. 21571886 10.1128/AEM.00421-11 PMC3127705

[26] Cho HJ (1983) Inactivation of the scrapie agent by pronase. Can J Comp Med 47: 494–496. 6230145 PMC1235983

[27] Tsiroulnikov K, Rezai H, Bonch-Osolovskaya E, Nedkov P, Gousterova A, et al (2004) Hydrolysis of the amyloid prion protein and non-pathogenic meat and bone meal by anaerobic thermophilic prokaryotes and Streptomyces subspecies. J Agri Food Chem 52: 6353–6360. 10.1021/jf0493324 15453713

[28] McLeod AH, Murdoch H, Dickinson J, Dennis MJ, Hall GA, et al (2004) Proteolytic inactivation of the bovine spongiform encephalopathy agent. Biochem Biophysic Res Comm 317: 1165–1170. 10.1016/j.bbrc.2004.03.168 15094392

[29] Jackson GS, Mckintosh E, Flechsig E, Prodromidou K, Hirsch P, et al (2005) An enzyme-detergent method for effective prion decontamination of surgical steel. J Gen Virol 86: 869–878. 15722550 10.1099/vir.0.80484-0

[30] Lawson VA, Stewart JD, Masters CL (2007) Enzymatic –detergent treatment protocol that reduces protease-resistant prion protein load and infectivity from surgical-steel monofilaments contaminated with a human derived prion strain. J Gen Virol 88: 2905–2914. 17872546 10.1099/vir.0.82961-0

[31] Croud VB, Collinge J, Jackson G (2008) Composition and methods for prion Decontamination. Unites States Patent Application Publication. US2008/0206843A1.

[32] Dickinson J, Murdoch H, Dennis MJ, Hall GA, Bott R, et al (2009) Decontamination of prion protein (BSE 301V) using genetically engineered protease. J Hosp Infect 72: 65–70. 19201054 10.1016/j.jhin.2008.12.007

[33] Vilette D, Andreoletti O, Archer F, Madelaine MF, Vilotte JL, et al (2001) Ex vivo propagation of infectious sheep scrapie agent in heterologous epithelial cells expressing ovine prion protein. Proc Natl Acad Sci USA 98: 4055–4059. 11259656 10.1073/pnas.061337998 PMC31178

[34] Hommel RK (1990) Formation and physiological role of biosurfactants produced by hydrocarbon–utilizing microorganisms. Biodegradation 1: 107–119. 1368144 10.1007/BF00058830

[35] Muthusamy K, Gopalakrishnan S, Ravi TK, Sivachidambaram P (2008) Biosurfactants: Properties, commercial production and application. Curr Sci 94: 736–747.

[36] Hisatsuka K, Nakahara T, Sano N, Yamada K (1971) Formation of rhamnolipid by *Pseudomonas aeruginosa* and its function in hydrocarbon fermentation. Agric Biol Chem 35: 686–692.

[37] Soberon-Chavez G, Lepine F, Deziel E (2005) Production of rhamnolipids by *Pseudomonas aeruginosa* . Appl Microbiol Biotechnol 68: 718–725. 16160828 10.1007/s00253-005-0150-3

[38] Desai JD, Banat IM (1997) Microbial production of surfactants and their commercial potential. Mol Biol Rev 61: 47–64. 10.1128/mmbr.61.1.47-64.1997 PMC232600 9106364

[39] Nitschke M, Coast SG (2007) Biosurfactants in food industry. Trends Food Sci Technol 18: 252–259.

[40] Wang Q, Fang X, Bai B, Liang X, Shuler PJ (2007) Engineering bacteria for production of rhamnolipid as an agent for enhanced oil recovery. Biotechnol Bioeng 98: 842–853. 17486652 10.1002/bit.21462

[41] Deziel E, Paquette G, Villemur R, Lepine F, Bisaillon J (1996) Biosurfactant production by a soil *pseudomonas* strain growing on polycyclic aromatic hydrocarbons. Appl Environ Microbial 62: 1908–1912. 10.1128/aem.62.6.1908-1912.1996 PMC1388868 16535330

[42] Kosaric N (2001) Biosurfactants and their application for soil remediation. Food Technol Biotechnol 39: 295–304.

[43] Noordman WH, Jassen DB (2002) Rhamnolipid stimulates uptake of hydrophobic compounds by *Psuedomonas aeruginosa* . Appl Environ Microbiol 68: 4502–4508. 12200306 10.1128/AEM.68.9.4502-4508.2002 PMC124127

[44] Cao X-H, Liao Z-Y, Wang C-L, Yang W-Y, Lu M-F (2009) Evaluation of a lipopeptide biosurfactant from *Bacillus natto* TK-1 as a potential source of anti-adhesive, antimicrobial and antitumor activities. Braz J Microbiol 40: 2373–379. 10.1590/S1517-838220090002000030 PMC3769733 24031375

[45] Rodrigues LR, Teixeira JA, van der mei HC, Oliveira R (2006) Isolation and partial characterization of a biosurfactant produced by *Streptococcus thermophilus* A. Colloids Surf. B. Biointerfaces 53: 105–112. 16987640 10.1016/j.colsurfb.2006.08.009

[46] Brzozowski B, Bednarski W, Golek P (2011) The adhesive capability of two *Lactobacillus* strains and physiochemical properties of their synthesised biosurfactants. Food Technol Biotechnol 49: 177–186.

[47] Singh A, Van Hamme JD, Ward OP (2007) Surfactants in microbiology and biotechnology. Part 2: Application aspects. Biotechnol Adv 25: 99–121. 17156965 10.1016/j.biotechadv.2006.10.004

[48] Piening N, Nonnon R, DiBari M, Walter S, Windl O, et al (2006) Conversion efficiency of bank cole prion preotein in vitro is determined by residues 155 and 170, but does not correlate with the high susceptibility of bank voles to sheep scrapie in vivo. J Biol Chem 281: 9373–9384. 16455657 10.1074/jbc.M512239200

[49] Mitsuiki S, Takasugi M, Moriyama Y, Futagami T, Golo M, et al (2010) Identification of alkaliphilic actinomycetes that produces a PrP^Sc^ degrading enzyme. Am Microbiol 60: 349–353.

[50] Okoroma EA, Garelick H, Abiola OO, Purchase D (2012) Identification and characterisation of a *Bacillus licheniformis* strain with profound keratinase activity for degradation of melanised feather. Int Biodeter Biodegra 74: 54–60.

[51] Zhang G, Wu Y, Qian X, Meng Q (2005) Biodegradation of crude oil by *Pseudomonas aeruginosa* in the presence of rhamnolipids. J Zhejiang Uni Science 6B 8: 725–730. 10.1631/jzus.2005.B0725 PMC1389852 16052704

[52] Neale MH, Mountjoy SJ, Edwards JC, Vilette D, Laude H, et al (2010) Infection of cell Lines with experimental and natural ovine scrapie agents. J Virol 84: 2444–2452. 20032176 10.1128/JVI.01855-09 PMC2820909

[53] Somerville RA, Oberthur RC, Havekost U, MacDonald F, Taylor DM, et al (2002) Characterization of thermodynamic diversity between transmissible spongiform encephalopathy agent strains and its theoretical implications. J Biol Chem 277: 11084–11089. 11792707 10.1074/jbc.M111766200

[54] Bolton DC, McKinley MP, Prusiner SB (1984) Molecular characterisation of the major scrapie prion protein. Biochemistry 23: 5898–5906. 6395885 10.1021/bi00320a002

[55] Oesch B, Jensen M, Nilsson P, Fogh J (1994) Properties of scrapie prion protein: quantitative analysis of protease resistance. Biochemistry 33: 5926–5931. 7910036 10.1021/bi00185a033

[56] Sharma R & Gupta R (2010) Substrate specificity characterisation of a thermostable keratinase from *Pseudomonas aeruginosa* KS-1. J Ind Microbiol Biotechnol 37: 785–792. 20414794 10.1007/s10295-010-0723-8

[57] Tork S, Aly MM, Nawar L (2010) Biochemical and molecular characterisation of a new local keratinase producing *Pseudomonas* sp. MS21. Asian J Biotechnol 2: 1–13.

[58] Ramnani P, Kumar SS, Gupta R (2005) Concomitant production and downstream processing of alkaline protease and biosurfactant from *Bacillus licheniformis* RG1: Bioformulation as detergent additive. Process Biochem 40: 3353–3359.

[59] Berardi VA, Cardone F, Valanzo A, Lu M, Pocchiari M (2006) Preparation of soluble infectious samples from scrapie-infected brain: a new tool to study the clearance of transmissible spongiform encephalopathy agents during plasma fractionation. Transfusion 46: 652–658. 16584444 10.1111/j.1537-2995.2006.00763.x

[60] Barron RM, Campbell SL, King D, Bellon A, Chapman KE, et al (2007) High titers of transmissible spongiform encephalopathy infectivity associated with extremely low levels of prp^Sc^ in vivo. J Biol Chem 282: 35878–35886. 17923484 10.1074/jbc.M704329200

[61] Lasmezas CI, Deslys JP, Robain O, Jaegly A, Beringue V, et al (1997) Transmission of the BSE agent to mice in the absence of detectable abnormal prion protein. Science 275: 402–405. 8994041 10.1126/science.275.5298.402

[62] Suzuki Y, Tsujimoto Y, Matsui H, Watanabe K (2006) Decomposition of extremely hard-to-degrade animal proteins by thermophilic bacteria. J Biosci 102: 73–81. 10.1263/jbb.102.73 17027867

[63] Pilon JL, Nash PB, Arver T, Hoglund D, Vercauteren KC (2009) Feasibility of infectious prion digestion using mild conditions and commercial subtilisin. J Virol Meth 161: 168–172. 10.1016/j.jviromet.2009.04.040 19467265

[64] Karanth NGK, Deo PG, Veenanadig NK (2005) Microbial production of biosurfactants and their importance. Cur Sci 77 (1): 116–126.

